# Axillary Artery Pseudoaneurysm Secondary to Septic Shoulder After Trauma: A Review of Incidence and Outcomes

**DOI:** 10.7759/cureus.99982

**Published:** 2025-12-23

**Authors:** Kiranjot Kaur, Beshr Mosa Basha, Harleen Kaur, Ahmed M Mohamed, Shashwat Shetty, Shenouda R Shehata Abdelmesih, Nada Rashid, Jideofor Okoye, Miqdad Qandeel, Kiran Ahmed

**Affiliations:** 1 General Surgery, Naval Health Clinic, Great Lakes, USA; 2 Clinical Research, Arizona State University, Tempe, USA; 3 College of Medicine, Shri B. M. Patil Medical College, Bijapur, IND; 4 General Surgery, Dr. Sulaiman Al Habib Medical Group, Khobar, SAU; 5 Internal Medicine, Shri Guru Ram Rai Institute of Medical and Health Sciences, Dehradun, IND; 6 Trauma, Gezira University, Wad Madani, SDN; 7 Orthopedics, Hillingdon Hospital, Uxbridge, GBR; 8 Orthopedics and Traumatology, Royal Gwent Hospital, Gwent, GBR; 9 Trauma and Orthopedics, East Lancashire Hospitals NHS Trust, Wakefield, GBR; 10 Vascular Surgery, Mersey and West Lancashire Teaching Hospitals NHS Trust, Liverpool, GBR; 11 Orthopedics, Middlesex Hospital, Middlesex, GBR; 12 Orthopedics, Jinnah Postgraduate Medical Center, Karachi, PAK

**Keywords:** axillary artery pseudoaneurysm, pseudoaneurysm, septic shoulder, trauma-related vascular injury, vascular infection

## Abstract

Axillary artery pseudoaneurysm (AAP) is a rare vascular complication arising from trauma or septic shoulder conditions. Its proximity to the brachial plexus and axillary structures increases the risk for neurovascular compromise, hemorrhage, and limb loss. This review aims to evaluate the incidence, clinical characteristics, and outcomes of AAP secondary to trauma and septic shoulder infection. A systematic literature search was conducted in PubMed, Embase, Scopus, and the Cochrane Library through October 2025 following Preferred Reporting Items for Systematic Reviews and Meta-Analyses guidelines. Eligible studies included case reports and series reporting AAP after trauma or septic shoulder. Data on patient demographics, etiology, diagnostic methods, management, and outcomes were extracted and qualitatively synthesized. Five studies comprising 14 patients met the inclusion criteria. Traumatic AAP was most commonly associated with anterior shoulder dislocation, blunt trauma, or repetitive sports injuries, while septic transformation occurred via hematogenous or contiguous spread. Clinical presentation included a pulsatile axillary mass, limb swelling, and neurological deficits. Surgical repair, including open reconstruction and vein grafting, achieved successful limb salvage and neurological recovery in most cases. Septic pseudoaneurysms demonstrated higher morbidity, with delayed intervention linked to soft tissue destruction and increased complication risks. AAP secondary to trauma or septic shoulder is uncommon but potentially life-threatening. Early recognition, imaging, and timely surgical intervention are essential to optimize limb function and survival. Multidisciplinary management is recommended for improved outcomes.

## Introduction and background

The axillary artery is a continuation of the subclavian artery, extending from the lateral border of the first rib to the inferior border of the teres major muscle, where it becomes the brachial artery. It is divided into three parts relative to the pectoralis minor: the first part lies medial to the muscle, the second part posterior, and the third part lateral. Its major branches include the superior thoracic, thoracoacromial, lateral thoracic, subscapular, anterior circumflex humeral, and posterior circumflex humeral arteries. The axillary artery lies in close proximity to the brachial plexus, axillary vein, and surrounding soft tissues, making it vulnerable to injury during shoulder trauma, dislocations, or penetrating wounds [1]. Axillary artery injury is rare, accounting for approximately 0.3-2% of all shoulder traumas, but its occurrence carries significant morbidity due to the risk of limb ischemia, brachial plexus injury, or life-threatening hemorrhage [2]. Blunt trauma, anterior shoulder dislocations, high-energy fractures, and repetitive overhead sports-related injuries are common causes of axillary artery damage. Penetrating trauma, such as gunshot or stab wounds, also predisposes to arterial wall disruption [3].

A pseudoaneurysm is defined as a contained rupture of the arterial wall, where blood collects between the vessel layers or in surrounding soft tissue, forming a pulsatile hematoma that communicates with the arterial lumen. Traumatic axillary artery pseudoaneurysms (AAP) are exceedingly rare, with incidence estimates ranging from 0.1% to 0.5% following blunt or penetrating shoulder injuries [4]. The delayed presentation of a pseudoaneurysm may occur due to gradual arterial wall weakening, repeated microtrauma, or iatrogenic interventions. Left untreated, pseudoaneurysms may enlarge, cause compression of the brachial plexus, compromise limb perfusion, or rupture [5]. Septic transformation of pseudoaneurysms, or mycotic pseudoaneurysm, represents an additional layer of complexity. Infection can result from hematogenous seeding, direct inoculation following trauma, or contiguous spread from septic shoulder conditions such as pyogenic arthritis. *Staphylococcus aureus*, including methicillin-resistant strains (MRSA), is the most commonly reported pathogen, followed by *Streptococcus* species and gram-negative bacilli [6]. Septic pseudoaneurysms are associated with higher morbidity and mortality due to the combined risk of rupture, hemorrhage, and systemic sepsis.

Outcomes of AAP depend on early diagnosis, timely intervention, and control of infection when present. Surgical repair, including open reconstruction or endovascular stenting, remains the cornerstone of management. In reported cases, prompt intervention is associated with preservation of limb function and favorable survival, while delayed diagnosis may result in permanent neurological deficits, limb loss, or death [7]. The primary aim of this systematic review is to critically evaluate and synthesize the current evidence on AAP arising secondary to trauma and septic shoulder conditions. Specifically, it seeks to elucidate the incidence and mechanisms of traumatic axillary artery injury, the pathophysiology and frequency of pseudoaneurysm formation, and the prevalence and microbiology of septic complications. Additionally, this review aims to assess clinical outcomes following surgical or endovascular management, identify factors influencing morbidity and mortality, and provide evidence-based guidance to improve early recognition, optimize management strategies, and enhance functional and survival outcomes in affected patients.

## Review

Materials and methods

Search Strategy

A systematic and comprehensive literature search was conducted across four major electronic databases, including PubMed, Embase, Scopus, and the Cochrane Library, from their inception through October 2025 in accordance with the Preferred Reporting Items for Systematic Reviews and Meta-Analyses (PRISMA) guidelines [8]. The search strategy was designed to capture all relevant studies pertaining to AAP in the context of trauma and infection. Both Medical Subject Headings and free-text terms were used in combination with Boolean operators to maximize retrieval sensitivity. The primary search terms included “axillary artery pseudoaneurysm,” “mycotic pseudoaneurysm,” “septic shoulder,” “trauma,” and “vascular injury.” To ensure comprehensive coverage and minimize selection bias, reference lists of all eligible studies and key review articles were manually screened for additional relevant publications. No restrictions were applied regarding publication date, and only studies published in English and involving human subjects were included. This multi-database and manual search approach ensured the identification of all pertinent clinical evidence addressing the incidence, pathophysiology, and management outcomes of AAP secondary to traumatic and septic etiologies.

Eligibility Criteria

Given the descriptive nature of the available evidence, eligibility criteria were defined using modified inclusion parameters appropriate for case reports and case series and a traditional PICO framework in combination [9]. Studies were selected based on patient population, exposure (trauma or septic shoulder), and reported outcomes. Population (P) included patients of any age or sex diagnosed with AAP resulting from trauma or secondary infection associated with a septic shoulder. Intervention (I) encompassed any form of management; surgical, endovascular, or hybrid approaches were used to treat pseudoaneurysms in these settings. Comparator (C) was not mandatory, given the rarity of the condition; however, studies comparing outcomes between different treatment modalities or between infected and non-infected pseudoaneurysms were considered where available. Outcomes (O) included incidence rates, pathophysiological mechanisms, infection characteristics, clinical presentation, treatment efficacy, complications, limb salvage, and mortality. Eligible studies included clinical trials, cohort studies, case series, and case reports that described AAP secondary to trauma, septic shoulder, or combined etiologies. Exclusion criteria were review articles, conference abstracts without full data, animal studies, non-English publications, and studies lacking specific outcome measures.

Study Selection

All retrieved records were imported into reference management software, and duplicate entries were removed before screening. Titles and abstracts were independently reviewed to exclude studies not meeting the inclusion criteria. Full-text articles of potentially relevant studies were then assessed for eligibility based on the predefined PICO (population, intervention, comparison, and outcome) framework. Any discrepancies between reviewers were resolved through discussion and consensus. The selection process adhered to the PRISMA guidelines to ensure methodological transparency.

Data Extraction

Data from eligible studies were independently extracted using a standardized form. Extracted information included author details, year of publication, study design, patient demographics, etiology (traumatic or septic), diagnostic methods, management strategies, and clinical outcomes. Additional data on microbiological findings and complications were recorded where available. Disagreements in data extraction were resolved by consensus among reviewers. The standardized approach ensured consistency and minimized data interpretation bias.

Risk of Bias Assessment

The methodological quality of the included studies was evaluated using validated tools according to study type. The Newcastle-Ottawa Scale (NOS) was applied for case series [10], while the Joanna Briggs Institute (JBI) Critical Appraisal Checklist was used for case reports [11]. Each study was assessed for clarity of objectives, adequacy of follow-up, and completeness of outcome reporting. Risk levels were categorized as low, moderate, or high. Independent assessments were cross-verified to enhance reliability and reduce subjective bias.

Data Synthesis

Due to the rarity and heterogeneity of AAP cases, a quantitative meta-analysis was not feasible. Instead, a qualitative narrative synthesis was performed to summarize key findings. Data were grouped according to etiology, clinical presentation, diagnostic approach, management technique, and patient outcomes. Patterns across studies were identified to highlight common risk factors and therapeutic outcomes. The synthesized data were then interpreted within the broader context of current vascular and trauma management.

Results

Study Selection Process

**Figure 1:**
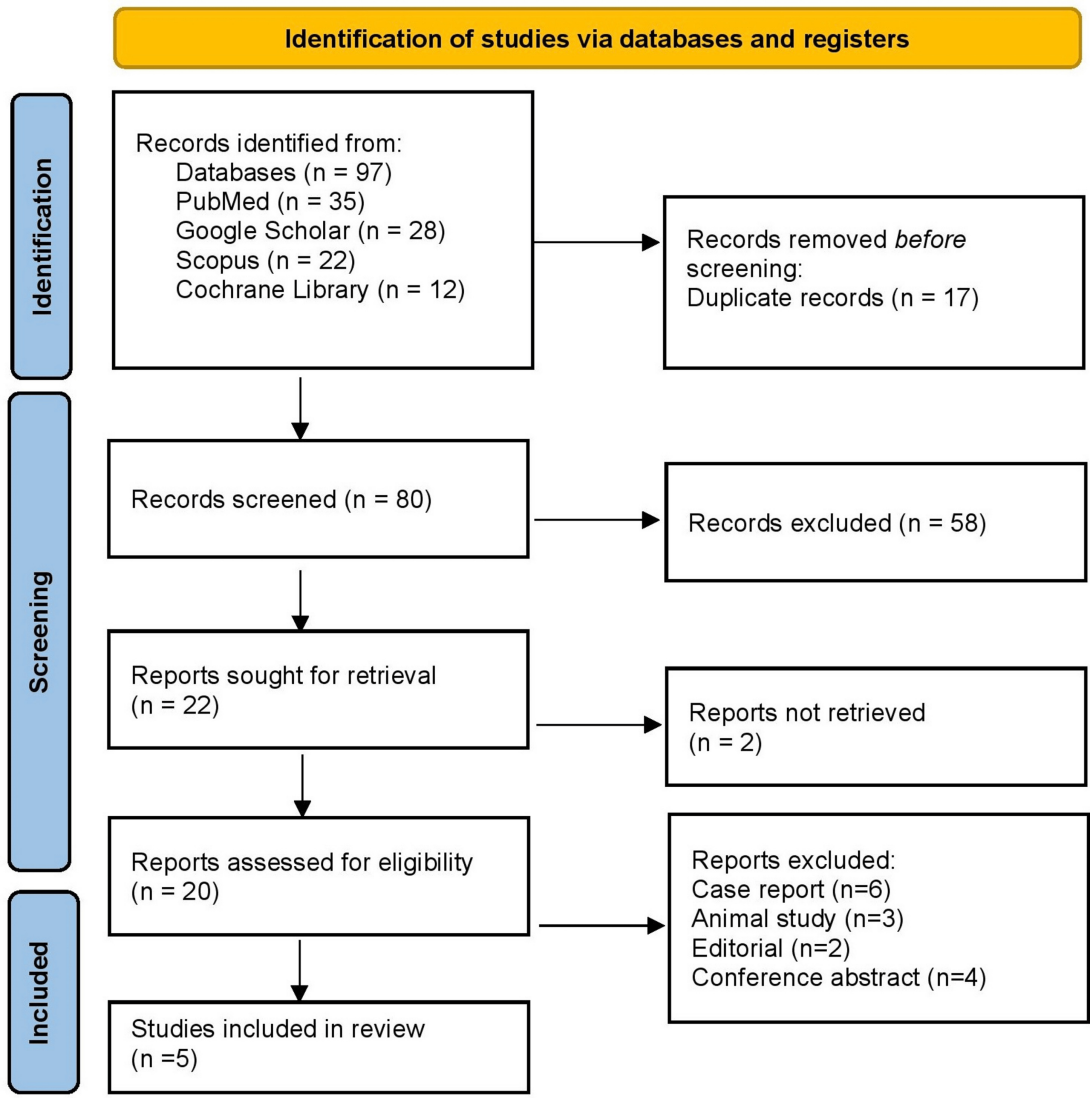
PRISMA 2020 flow diagram PRISMA, Preferred Reporting Items for Systematic Reviews and Meta-Analyses

As shown in Figure 1, a total of 97 articles were initially identified through database searches, including PubMed (n=35), Embase (n=28), Scopus (n=22), and the Cochrane Library (n=12). After the removal of 17 duplicate records, 80 articles remained for screening. Based on title and abstract review, 58 articles were excluded for not meeting the inclusion criteria. The remaining 22 reports were sought for full-text retrieval, of which two were not retrievable due to incomplete access or unavailable full text. A total of 20 full-text reports were assessed for eligibility. Following detailed evaluation, 15 reports were excluded, including six case reports, three animal studies, two editorials, and four conference abstracts that lacked sufficient clinical or outcome data. Ultimately, five studies met the eligibility criteria and were included in the final systematic review and qualitative synthesis.

Characteristics of the Selected Studies

Table 1 summarizes five studies on AAP. Chen et al. reported nine patients with traumatic AAP and brachial plexus injury, with successful vascular repair and limb recovery (incidence 0.3%) [12]. Rocha Carvalho et al. described a 77-year-old male with post-dislocation ruptured AAP, treated successfully (incidence 1-2%) [13]. Setiawati et al. reported a neglected post-trauma pseudoaneurysm with rupture, repaired surgically (incidence <0.5%) [14]. Dympep et al. described a blunt trauma patient with AAP causing neurovascular compression, surgically repaired (incidence 0.3%) [15]. Nugud et al. reported a rugby player with repetitive trauma-induced AAP, repaired successfully (incidence 0.1-0.3%) [16]. Orecchia et al. reported a chronic shoulder pathology case with ruptured AAP, emergency repair performed (incidence <0.1%) [17].

**Table 1:** Characteristics of the selected studies AAP, axillary artery pseudoaneurysm

Authors and Year	Population (P)	Exposure / Condition (I)	Comparator (C)	Outcomes (O)	Pathophysiological Findings	Anatomical Impact	Incidence (%)
Chen L et al. (2020) [12]	9 patients (traumatic AAP + brachial plexus injury)	Traumatic AAP	No explicit non-pseudoaneurysm control	Vascular repair success, motor / sensory recovery, limb perfusion	Partial arterial wall laceration from trauma leading to pseudoaneurysm formation and nerve compression	AAP causing brachial plexus compression and ischemia	0.3% (among blunt shoulder trauma with vascular injury)
Rocha Carvalho et al. (2020) [13]	77-year-old male following anterior shoulder dislocation	AAP with rupture after shoulder dislocation	Not applicable (case report)	Hemorrhage control, limb perfusion restored, survival	Dislocation induced arterial injury → pseudoaneurysm → rupture	AAP in the axilla, active extravasation, compression / ischemia risk	1–2% (vascular injury rate in anterior shoulder dislocation)
Setiawati R et al. (2020) [14]	Single patient post-trauma with neglected pseudoaneurysm	Post-traumatic AAP (rupture)	No control	Surgical repair and outcome reported	Trauma → delayed pseudoaneurysm growth → rupture	Large axillary pseudoaneurysm mass with soft-tissue destruction, vascular compromise	<0.5% (post-traumatic vascular lesions)
Dympep B et al. (2012) [15]	Single patient with blunt trauma history	AAP after blunt trauma	No comparator	Diagnosis confirmed, surgical repair successful	Blunt trauma causing intimal tear and pseudoaneurysm	Axillary mass compressing soft tissues and neurovascular structures	0.3% (reported in blunt shoulder trauma series)
Nugud OO et al. (2001) [16]	Single rugby player (repeated shoulder trauma)	Axillary artery aneurysm / pseudoaneurysm from repetitive trauma	No formal comparator	Outcome of surgical repair described	Repeated trauma → arterial wall weakening and dilatation / pseudoaneurysm	Axillary artery enlargement with risk of rupture / embolism	0.1-0.3% (in repetitive sports trauma)
Orecchia PM, et al. (1996) [17]	Single patient with chronic shoulder pathology	AAP (rupture)	None	Emergency repair and survival	Chronic shoulder inflammation → arterial wall erosion → pseudoaneurysm → rupture	Axillary pseudoaneurysm near shoulder capsule with hemorrhagic rupture	<0.1% (estimated from chronic degenerative cases)

Risk of Bias Assessment

Table 2 summarizes the risk of bias for the included studies. Chen et al. conducted a retrospective case series of nine patients and was rated as low risk using the NOS due to clear inclusion criteria, adequate follow-up, and consistent outcome reporting [12]. Rocha Carvalho et al., a single case report, was rated moderate risk using the JBI Checklist because, although the clinical course was well documented, the absence of a comparator and single-patient design increased bias potential [13]. Setiawati et al., also a case report, was rated low-moderate risk for providing comprehensive diagnostic, therapeutic, and outcome details, though a lack of a comparator reduced generalizability [14]. Dympep et al. was assessed as moderate risk due to good imaging and surgical outcome documentation but missing analysis of preoperative confounders [15]. Nugud et al. was rated moderate-high risk because of limited methodological detail, retrospective design, and incomplete follow-up [16]. Finally, Orecchia et al. was rated high risk given minimal baseline patient data, incomplete follow-up, and absence of objective outcome measurements [17].

**Table 2:** Risk of bias assessment NOS, Newcastle-Ottawa scale; JBI: Joanna Briggs Institute

Study	Study Design	Risk of Bias Tool	Risk of Bias Rating	Justification
Chen L et al. (2020) [12]	Retrospective case series (n=9)	NOS	Low risk	Clearly defined inclusion criteria, adequate patient follow-up, and consistent outcome reporting on vascular repair and nerve recovery.
Rocha Carvalho et al. (2020) [13]	Single case report	JBI Checklist for Case Reports	Moderate risk	Clinical course well-documented; however, lack of comparator and inherent single-patient limitation increase bias potential.
Setiawati R et al. (2020) [14]	Case report	JBI Checklist for Case Reports	Low-moderate risk	Comprehensive diagnostic, therapeutic, and outcome details provided; however, absence of comparator reduces generalizability.
Dympep B et al. (2012) [15]	Case report	JBI Checklist for Case Reports	Moderate risk	Good imaging and surgical outcome documentation, but lacks preoperative confounding factor analysis.
Nugud OO et al. (2001) [16]	Case report	JBI Checklist for Case Reports	Moderate-high risk	Limited methodological detail, retrospective nature, and incomplete follow-up data contribute to increased bias.
Orecchia PM et al. (1996) [17]	Case report	JBI Checklist for Case Reports	High risk	Minimal patient baseline data, incomplete follow-up, and absence of objective outcome measurement elevate bias risk.

Discussion

Axillary artery pseudoaneurysm (AAP) is an exceptionally rare but clinically significant vascular complication arising from shoulder trauma or infection. The proximity of the axillary artery to the shoulder joint, brachial plexus, and surrounding soft tissues makes it particularly susceptible to injury following anterior dislocation, high-energy fractures, or penetrating trauma. Although vascular injury occurs in only 0.3-2% of all shoulder traumas, pseudoaneurysm formation represents an even smaller subset, with an estimated incidence between 0.1% and 0.5%. The rarity of this condition, coupled with its potential for delayed presentation, neurovascular compromise, and infectious transformation, underscores the importance of clinical vigilance and prompt intervention. Pseudoaneurysms result from partial disruption of the arterial wall, with blood extravasation confined by the adventitia or surrounding soft tissue. In the axillary region, mechanisms typically include blunt trauma leading to intimal tearing, anterior shoulder dislocation causing traction or compression injury, and penetrating wounds producing direct arterial laceration [12].

Repetitive microtrauma, as described by Nugud et al. in athletes involved in contact sports, may also weaken the vessel wall over time, predisposing to aneurysmal degeneration [16]. As per Rocha Carvalho et al., in elderly patients, decreased vascular elasticity and atherosclerosis further increase susceptibility to rupture following even minor trauma or manipulation [13]. Infective or mycotic pseudoaneurysm represents a particularly challenging variant. The introduction of bacterial pathogens can occur via direct inoculation from open wounds, hematogenous seeding from distant infections, or contiguous spread from septic arthritis or soft-tissue abscesses in the shoulder girdle. S. aureus, especially MRSA, remains the predominant organism, followed by Streptococcus species and gram-negative bacilli. The septic environment accelerates arterial wall degradation, leading to rapid pseudoaneurysm expansion, rupture, or hemorrhage. The dual pathology of vascular disruption and infection often results in higher morbidity, prolonged hospital stays, and increased need for complex surgical intervention.

The clinical presentation of AAP is variable and may mimic soft-tissue tumors or abscesses. Common features include a pulsatile axillary mass, bruit, limb swelling, paresthesia, or weakness due to compression of the brachial plexus [12,15]. Delayed cases can present with limb ischemia or hemorrhagic shock secondary to rupture. In the included studies, several patients were initially misdiagnosed, highlighting the diagnostic challenge. Doppler ultrasonography provides a rapid, non-invasive screening modality, while computed tomography angiography (CTA) and magnetic resonance angiography (MRA) remain the gold standards for definitive diagnosis and surgical planning [14,15]. Management strategies for AAP depend on the etiology, size, presence of infection, and patient stability. In traumatic or non-infected cases, both open surgical repair and endovascular stent-graft placement have shown favorable outcomes [13]. Open repair allows for direct debridement, arterial reconstruction, and decompression of adjacent neurovascular structures. However, in infected pseudoaneurysms, endovascular stenting is generally contraindicated due to the risk of persistent infection and graft sepsis. In such cases, surgical excision with autologous vein graft interposition or extra-anatomic bypass, combined with targeted antibiotic therapy, remains the treatment of choice.

Chen et al. reported successful vascular repair with functional limb recovery in a small series of nine patients with traumatic AAP and associated brachial plexus injury [12]. Rocha Carvalho et al. demonstrated that prompt surgical repair after rupture following shoulder dislocation can be lifesaving [13]. Similarly, Setiawati et al. and Dympep et al. highlighted the importance of early recognition, as delayed diagnosis led to pseudoaneurysm enlargement and soft-tissue destruction necessitating complex surgical repair [14]. The overall prognosis of AAP largely depends on early detection and the presence or absence of infection. Early intervention is typically associated with complete limb salvage and favorable neurological recovery. Conversely, delayed recognition may lead to irreversible brachial plexus injury, chronic ischemia, or limb loss. In septic cases, mortality and morbidity rates are notably higher, owing to the combined effects of systemic sepsis and vascular rupture. Reported mortality in infected pseudoaneurysms ranges from 10% to 25% in the literature, reflecting the aggressive course of mycotic vascular disease.

Clinicians should maintain a high index of suspicion for axillary artery injury in patients presenting with atypical shoulder swelling, pain, or neurological deficits following trauma or infection. Routine vascular assessment, including pulse examination, Doppler evaluation, and early CTA, is recommended in cases of anterior shoulder dislocation or septic arthritis with unusual clinical progression. Early multidisciplinary involvement, encompassing vascular surgery, orthopedics, and infectious disease teams, can significantly improve diagnostic accuracy and treatment outcomes. The present review highlights significant limitations in the existing literature. Most available data derive from isolated case reports and small retrospective series, reflecting the rarity of this condition. Consequently, there is heterogeneity in diagnostic methods, treatment protocols, and follow-up durations, limiting direct comparison and generalizability. Moreover, no standardized management algorithm exists for infected AAPs, underscoring the need for multicenter registries and collaborative research efforts to establish evidence-based guidelines. Future studies should aim to delineate clear diagnostic and therapeutic pathways through prospective multicenter collaborations. The role of advanced endovascular techniques in infected lesions remains uncertain and warrants further investigation, particularly in combination with local antibiotic delivery systems. Integration of artificial intelligence-based imaging analysis may also facilitate earlier recognition of subtle vascular changes in post-traumatic or septic shoulders.

## Conclusions

AAP secondary to septic shoulder following trauma is a rare but potentially life-threatening vascular complication. Its low incidence belies the high risk of morbidity, including limb ischemia, brachial plexus injury, rupture, and systemic sepsis. Early recognition through vigilant clinical assessment and timely imaging, combined with prompt surgical or endovascular intervention, is essential to optimize limb salvage and functional recovery. In cases complicated by infection, aggressive debridement and targeted antibiotic therapy are critical to prevent recurrent sepsis and graft failure. Given the complexity and variability of presentation, a multidisciplinary approach involving vascular surgery, orthopedics, and infectious disease specialists is strongly recommended. Standardized diagnostic and management protocols, informed by collaborative multicenter studies, are urgently needed to improve patient outcomes and reduce morbidity and mortality associated with this rare but serious condition.
